# Microstructure analysis of low electron density contrast metallic multilayers using resonant X-ray reflectivity

**DOI:** 10.1107/S1600577526001827

**Published:** 2026-04-02

**Authors:** P. N. Rao, M. K. Swami, H. Srivastava, P. Sahu, A. Ghosh, S. K. Rai

**Affiliations:** a Accelerator Physics and Synchrotrons Utilization Division Raja Ramanna Centre for Advanced Technology Indore452013 India; b Homi Bhabha National Institute Anushakti Nagar Mumbai400094 India; Bhabha Atomic Research Centre, India

**Keywords:** metallic multilayers, electron density, interfaces, resonant X-ray reflectivity

## Abstract

Resonant X-ray reflectivity (RXRR) was employed to investigate the microstructural properties, such as layer thicknesses, densities and interface widths, of metallic multilayers with low electron density contrast. Cu/Nb multilayers were chosen as a model system to demonstrate the enhanced sensitivity of RXRR. These multilayers, consisting of ten bilayers with varying period thicknesses, were deposited using magnetron sputtering. X-ray reflectivity measurements were performed at the *K*-absorption edges of both Cu and Nb, as well as using conventional Cu *K*α radiation. A pronounced difference was observed between reflectivity profiles acquired at these different energies. The enhanced contrast near the absorption edges enabled an effective means to extract microstructural information. The results demonstrate that RXRR is a powerful technique for characterizing metallic multilayers with low electron density contrast.

## Abstract 2

Resonant X-ray reflectivity was employed to probe the interfaces of low electron density contrast metallic multilayers.

## 1. Introduction

Nanoscale metallic multilayers (NMMs) consist of two or three alternating single-phase metallic layers with individual layer thicknesses in nanoscale dimension (Artz, 1998 ▸; Sáenz-Trevizo & Hodge, 2020 ▸; Zhou *et al.*, 2015 ▸). NMMs exhibit a wide range of functional properties, including high mechanical strength, fracture toughness, radiation resistance, and enhanced optical, electrical, magnetic responses and thermal behaviours (Niu *et al.*, 2012 ▸; Misra *et al.*, 2005 ▸; Misra & Krug, 2001 ▸; Misra *et al.*, 2004 ▸; Misra & Hoagland, 2005 ▸; Mara *et al.*, 2008 ▸; Ma *et al.*, 2017 ▸). As layer thickness approaches the nanoscale dimension there exists a large surface-to-volume ratio and the presence of multiple interfaces in NMMs, which contribute to their exceptional performance.

The deformation mechanism in NMMs is typically categorized into three regimes depending on the layer thickness (*d*). First is the Hall–Petch regime for *d* ≥ 75 nm, second is the single-dislocation regime for 5 nm ≤ *d* ≤ 75 nm and third is the interface-crossing regime for *d* ≤ 5 nm (Sáenz-Trevizo & Hodge, 2020 ▸; Zhou *et al.*, 2015 ▸; Misra *et al.*, 2005 ▸; Cui *et al.*, 2019 ▸). Mechanical strength in NMMs typically increases as the individual layer thickness decreases, but may decrease when the individual layer thickness is reduced below ∼1 nm due to interface-mediated softening mechanisms (Zhou *et al.*, 2015 ▸; Misra *et al.*, 2005 ▸, 1999 ▸; Fu *et al.*, 2008 ▸). In optical applications, especially in the extreme ultraviolet and soft X-ray regions, both peak reflectivity and bandwidth are influenced by the layer thickness ratio and interface roughness (Spiller, 1994 ▸; Xu *et al.*, 2015 ▸; Huang *et al.*, 2017 ▸). The interface width must typically be less than 10% of the bi-layer period. Therefore, precise control over layer and interface dimensions is essential for optimizing performance.

Magnetic properties such as giant magnetoresistance, perpendicular magnetic anisotropy, and magnetic skyrmions in NMMs are also strongly influenced by layer thickness and interface roughness (Tsymbal & Pettifor, 2001 ▸; Johnson *et al.*, 1996 ▸; den Broeder *et al.*, 1991 ▸). The strength and nature of exchange coupling in NMMs depend on the thickness of non-magnetic layers and interfaces characteristics (Bakonyi & Péter, 2010 ▸; Gijs & Bauer, 1997 ▸; Rizal *et al.*, 2016 ▸). For example, in Fe/Cr multilayers, increased interface roughness has been shown to reduce giant magnetoresistance nonlinearly (Gupta *et al.*, 2000 ▸). Thermal stability is crucial for maintaining and extending the functional performance of NMMs (Ma *et al.*, 2017 ▸; Andrievski, 2014 ▸; Kucharska *et al.*, 2012 ▸; Zeng *et al.*, 2017 ▸). The smaller dimension of layer thickness and large driving forces make interdiffusion occur at a faster time scale and cause the lower thermal stability of NMMs. For example, Cu/Nb multilayers with layer thickness greater than 35 nm exhibited long-term thermal stability (Misra *et al.*, 2005 ▸). Tailoring the length scales of layers and interfaces provides insight into the design of NMMs for numerous applications. In addition, the properties of NMMs further depend on, but are not limited to, the grain boundaries, grain size, texture, morphology, layer composition and deposition methods (Sáenz-Trevizo & Hodge, 2020 ▸; Callisti & Polcar, 2017 ▸; Wang & Misra, 2011 ▸).

To characterize the microstructure and composition of NMMs, various analytical techniques are employed (Russell, 1990 ▸). In general, layer thickness and interface roughness are determined using grazing incidence hard X-ray reflectivity (GIXRR), neutron reflectivity, and transmission electron microscopy (TEM) in cross-sectional mode. However, each technique has limitations. TEM provides highly localized structural information but is destructive in nature, preventing repetitive measurements on the same sample. Neutron reflectivity often suffers from a low signal-to-noise ratio and a limited scattering-vector range, leading to large errors (Wang *et al.*, 2005 ▸). GIXRR, though non-destructive and widely accessible, depends on electron density contrast between layers, which can be very low in many metallic multilayers, making the technique less effective.

To address the contrast limitation in GIXRR, resonant X-ray reflectivity (RXRR) is employed, where the incident X-ray energy is tuned near the absorption edge of one of the constituent elements (Zhernenkov *et al.*, 2014 ▸; Kemik *et al.*, 2011 ▸). In this study, we investigate Cu/Nb multilayers, which exhibit only ∼2% electron density contrast at Cu *K*α radiation, using RXRR to enhance sensitivity in probing layer thickness and interface quality.

## 2. Resonant hard X-ray reflectivity

In the X-ray regime the complex refractive index of a material can be expressed as (Spiller, 1994 ▸)
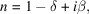
where
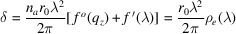
and
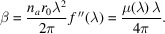
The term δ is the dispersion coefficient and β is the absorption coefficient, which are also known as optical indices; *r*_0_ is the classical electron radius; ρ_e_ (λ) and μ are wavelength-dependent electron density and the mass absorption coefficient, respectively; *f*^ *o*^(*q*_*z*_) is the Thomson atomic scattering factor (ASF) due to free electrons; and *f* ′(λ) and *f* ′′(λ) are the dispersion and absorption corrections to the ASF that arise from the bounded electrons in an atom. The contrast between two elements in a multilayer can be defined in terms of either optical contrast (Δδ^2^ + Δβ^2^) or electron density contrast (Russell, 1990 ▸; Rao *et al.*, 2019 ▸). In the extreme ultraviolet to soft X-ray regime (0.6 nm < λ < 41 nm), absorption plays a significant role and reflectivity is primarily governed by optical contrast, whereas, in the hard X-ray regime (λ < 0.6 nm), absorption is relatively weak and reflectivity is dominated by electron density contrast between the elements. Since the *K*-absorption edges of most metallic elements lie within the hard X-ray regime, contrast between elements in reflectivity is typically defined in terms of electron density. The electron density contrast between two elements can be expressed as
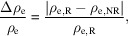
where ρ_e,R_ and ρ_e,NR_ are electron densities of the resonating and non-resonating atoms, respectively. In a multilayer system, when the incident energy is near the *K*-edge of one of the elements, that element is termed the resonating atom, while the other is the non-resonating atom. We have calculated the electron density contrast for selected metallic multilayer systems at Cu *K*α radiation and at the *K*-edges of the respective elements and present it in Table 1 ▸. The electron densities were derived from the optical constants measured and tabulated by Henke *et al.* (1993 ▸).

**Figure 1:**
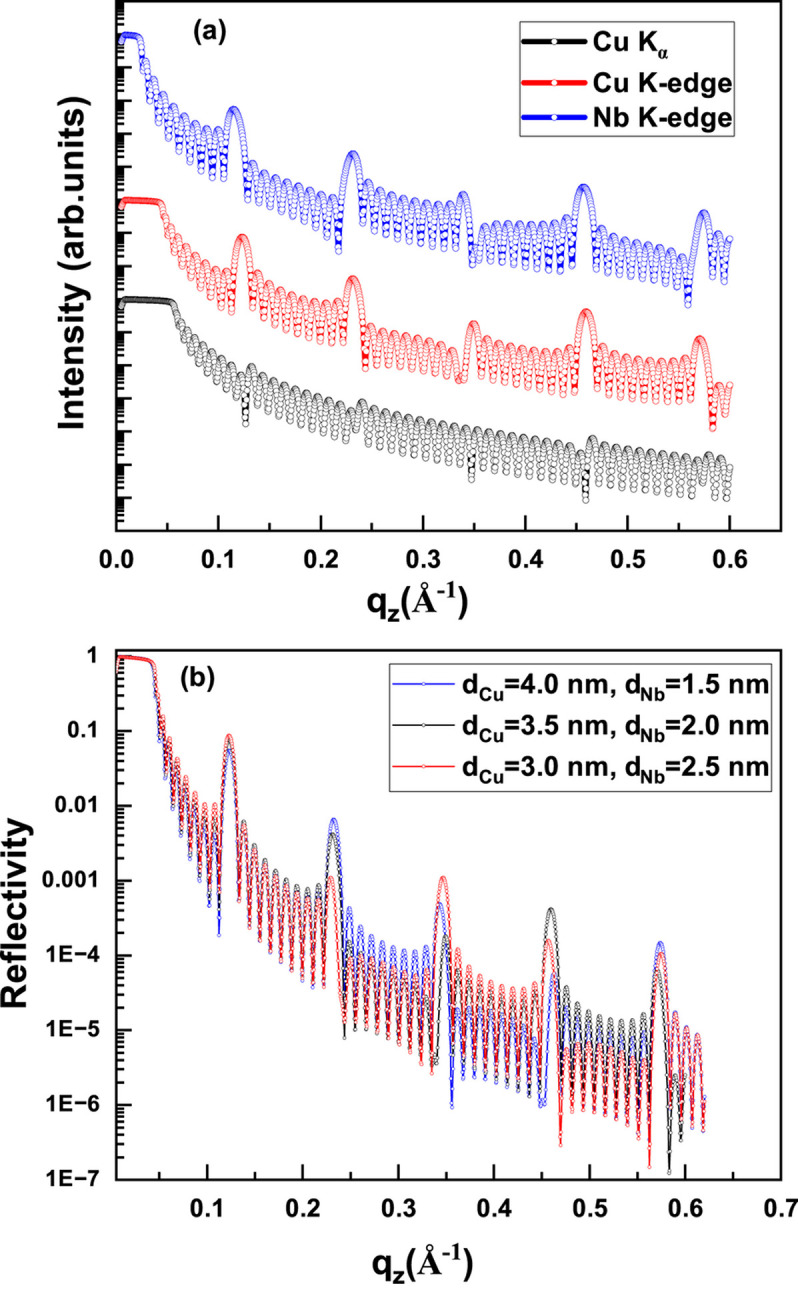
(*a*) The simulated reflectivity of an Si/[Cu (3.5 nm)/Nb (2.0 nm)]_*N*=10_ multilayer at Cu *K*α (*E* = 8048 eV), the Cu *K*-absorption edge (*E* = 8980.5 eV) and Nb *K*-absorption edge (*E* = 18983.0 eV), where *N* is the number of layer pairs. The curves are shifted vertically for better clarity. (*b*) Sensitivity of RXRR to Cu and Nb layer thickness variations at the Cu *K*-absorption edge.

As shown in Table 1 ▸, tuning the photon energy to an absorption edge significantly improves electron density contrast. This is further illustrated in Fig. 1 ▸(*a*), which shows simulated reflectivity curves of Cu/Nb multilayers consisting of ten layer pairs as a function of scattering vector, *q*_*z*_ [= 4π sin (θ)/λ, where θ is the grazing incidence angle and λ is the wavelength of the probing beam], at Cu *K*α radiation and *K*-absorption edges of Cu and Nb. Simulations were performed using *REFLEX* software (Vignaud & Gibaud, 2019 ▸) under ideal conditions, assuming sharp interfaces without roughness.

The primary difference among the simulated reflectivity profiles is the emergence of prominent Bragg peaks near the respective *K*-absorption edges. The reflection at each interface increases because of the enhanced electron density contrast, leading to the appearance of these pronounced Bragg features. Such reflectivity profiles show enhanced sensitivity to structural parameters such as layer thickness, interface width and electron density, which results in reliable estimation of these parameters. The sensitivity to changes in the layer thickness ratio is illustrated in Fig. 1 ▸(*b*), using simulations performed near the Cu *K*-edge. Variations in Bragg peak intensity and position were observed with nanometre-scale changes in layer thicknesses. A similar sensitivity was obtained in simulations conducted near the Nb *K* edge, demonstrating the broad applicability of this method. Thus, reflectivity measurements performed under enhanced electron density contrast provide an effective means to extract the structural information from NMMs with low electron density contrast.

## 3. Experimental details

Cu/Nb multilayer samples were deposited on *p*-type doped Si (100) substrates using a magnetron sputtering system (Rao *et al.*, 2024 ▸, 2025 ▸). Prior to deposition, the Si substrates were ultrasonically cleaned using iso­propyl alcohol. High-purity 2-inch Cu (99.999%) and Nb (99.95%) targets were used for sputtering. The deposition chamber was evacuated to a base pressure of 3 × 10^−5^ Pa before initiating the process. Deposition was carried out using high-purity (99.999%) argon gas, with a constant flow rate of 7 sccm (standard cubic centimetres per minute) controlled via a mass flow controller. During deposition, the chamber pressure was maintained at 0.25 Pa, and the distance between the targets and substrate was set to ∼100 mm. The deposition rates for Cu and Nb were calibrated by depositing individual thin films of each material before the multilayer fabrication. The Cu/Nb multilayers were deposited with rates of 0.045 nm s^−1^ for Cu and 0.065 nm s^−1^ for Nb. Two Cu/Nb multilayers with period thicknesses of 9.0 nm (ML-1) and 6.0 nm (ML-2) consisting of ten layer pairs were deposited for the present study. GIXRR measurements at Cu *K*α radiation were performed using a Bruker D8 Discover diffractometer. Additional reflectivity measurements near the *K*-absorption edges were carried out at beamline BL-02 of the Indus-2 synchrotron radiation source (Gupta *et al.*, 2021 ▸). The experimental reflectivity curves were initially fitted using the Parratt recursive formalism (Parratt, 1954 ▸), which is a nonlinear least-square curve fitting technique based on χ^2^ minimization, where χ^2^ represents the goodness of fit. Cross-sectional TEM (CXTEM) was performed using a Thermo Fisher Scientific Talos F200X G2 instrument, operated at an accelerating voltage of 200 kV, while distribution of elements were analysed through energy-dispersive X-ray spectroscopy (EDS).

## 4. Results and discussion

**Figure 2:**
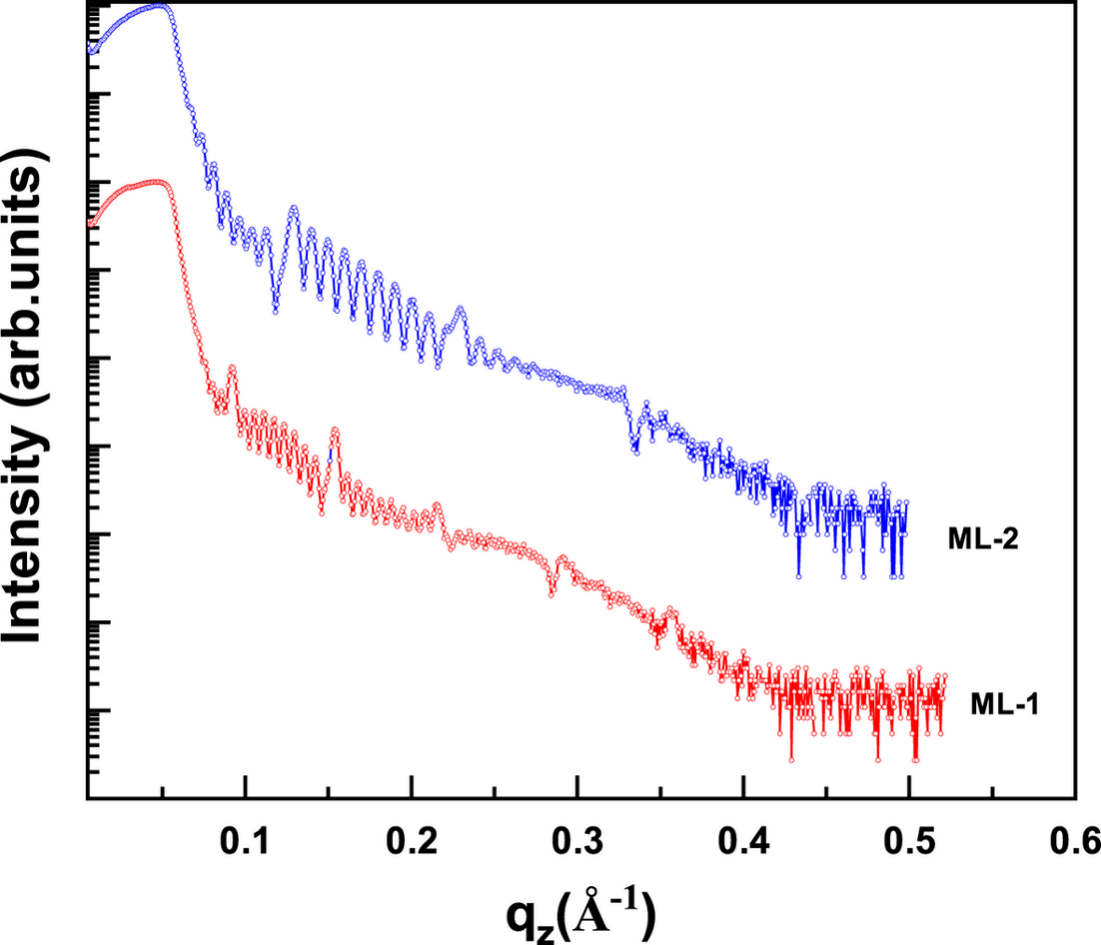
The measured reflectivity curves of Cu/Nb multilayer samples ML-1 and ML-2 at a Cu *K*α (*E* = 8048 eV) X-ray source.

The measured reflectivity curves of Cu/Nb multilayer samples ML-1 and ML-2, each consisting of ten layer pairs with different individual layer thicknesses deposited on Si substrate at a Cu *K*α X-ray source, are shown in Fig. 2 ▸. The reflectivity curves show *N*^−2^ Kiessig oscillations, where *N* is the number of layer pairs, arising from the total thickness of the multilayer stack, followed by first- and higher-order Bragg peaks. Due to the low electron density contrast between Cu and Nb, reflection at the interface is weak, resulting in less prominent Bragg peaks and making it difficult to infer structural information. Consequently, fitting the reflectivity curves using structural models with different Cu and Nb thicknesses may yield similar results leading to significant uncertainties in estimating layers thickness and interfaces roughness.

**Figure 3:**
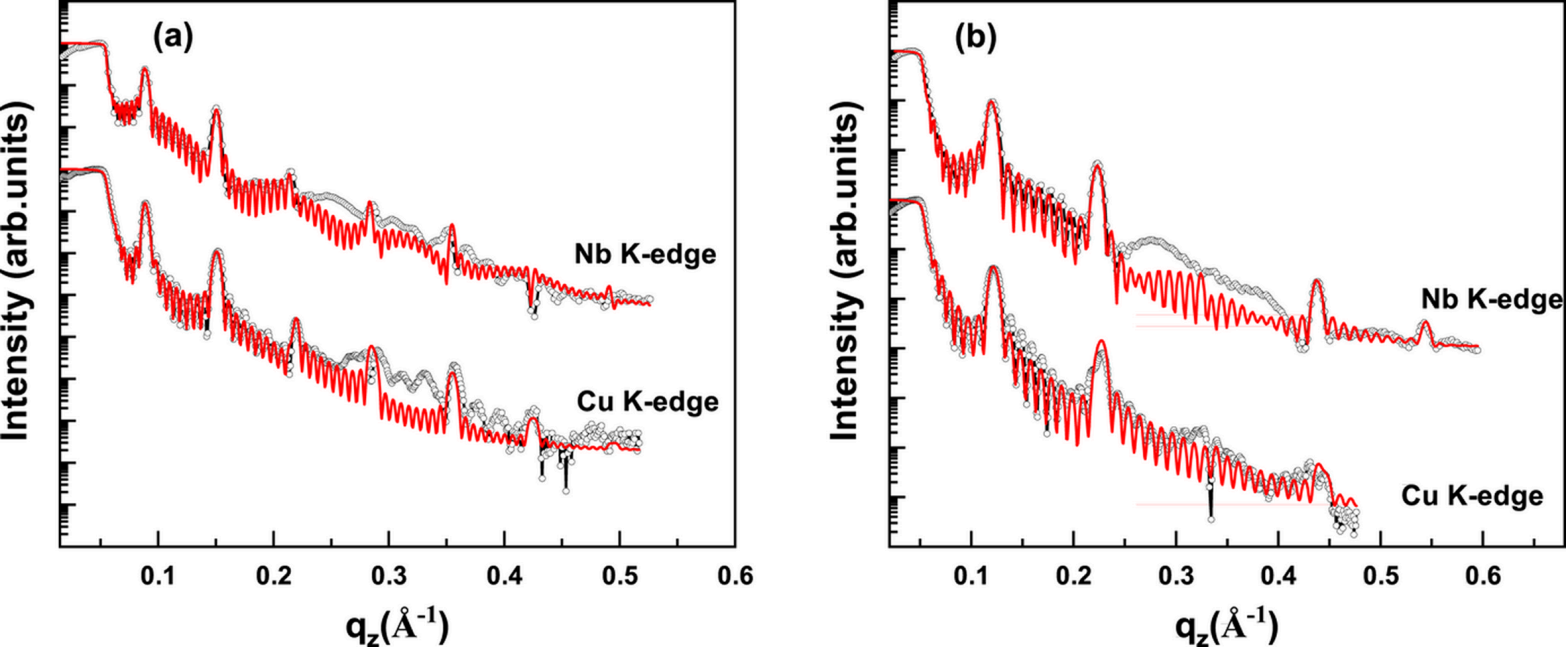
The measured (open circle) and simulated (continuous line) RXRR curves of Cu/Nb multilayer samples (*a*) ML-1 and (*b*) ML-2 at Cu and Nb *K*-absorption edges. The curves are shifted vertically for better clarity.

To address this contrast limitation, reflectivity measurements were also performed near the Cu and Nb *K*-absorption edges. The measured and simulated reflectivity curves of Cu/Nb multilayer samples ML-1 and ML-2 at these absorption edges are shown in Fig. 3 ▸. A key distinction from the measurements using the laboratory Cu *K*α source is the appearance of significantly stronger Bragg peaks at the absorption edges, owing to enhanced electron density contrast.

For structural modelling, a simple two-layer periodic model consisting of alternating Cu and Nb layers was assumed. The multilayer structure was represented by repeating this bilayer unit. During the fitting process, the layers thickness was estimated from the deposition rates, and tabulated electron density values were used as starting parameters. The fitting procedure minimized the difference between the measured and simulated reflectivity curves by adjusting the Cu and Nb layers thickness (*d*), electron densities (ρ_e_), and interfaces roughness for both the Cu-on-Nb and Nb-on-Cu interfaces. Imperfections at the interfaces reduce the reflectivity and are accounted for in the reflectivity analysis through a Debye–Waller-like damping factor, 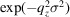, where σ denotes the root-mean-square interface roughness. A contaminated layer on top of the multilayer stack was also included in the model. To fit the reflectivity data of the Cu/Nb multilayer sample ML-2, a symmetric interface layer model is assumed. This assumption is based on the absence of the third-order Bragg peak in the reflectivity profile. In multilayer reflectivity, the intensity of Bragg peaks is modulated by a structure factor, 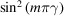, where γ is the ratio of thickness of non-resonating material to bilayer period and *m* is the order of the Bragg peak (Spiller, 1988 ▸). A particular Bragg peak is suppressed when the product *m*πγ is equal to, or is an integer multiple of, π due to phase cancellation of reflections from the adjacent interfaces within a bilayer. Such exact phase cancellation occurs only for symmetric interfaces. In the presence of asymmetric interfaces, the cancellation is incomplete, leading to the reappearance of the otherwise forbidden Bragg peak.

**Figure 4:**
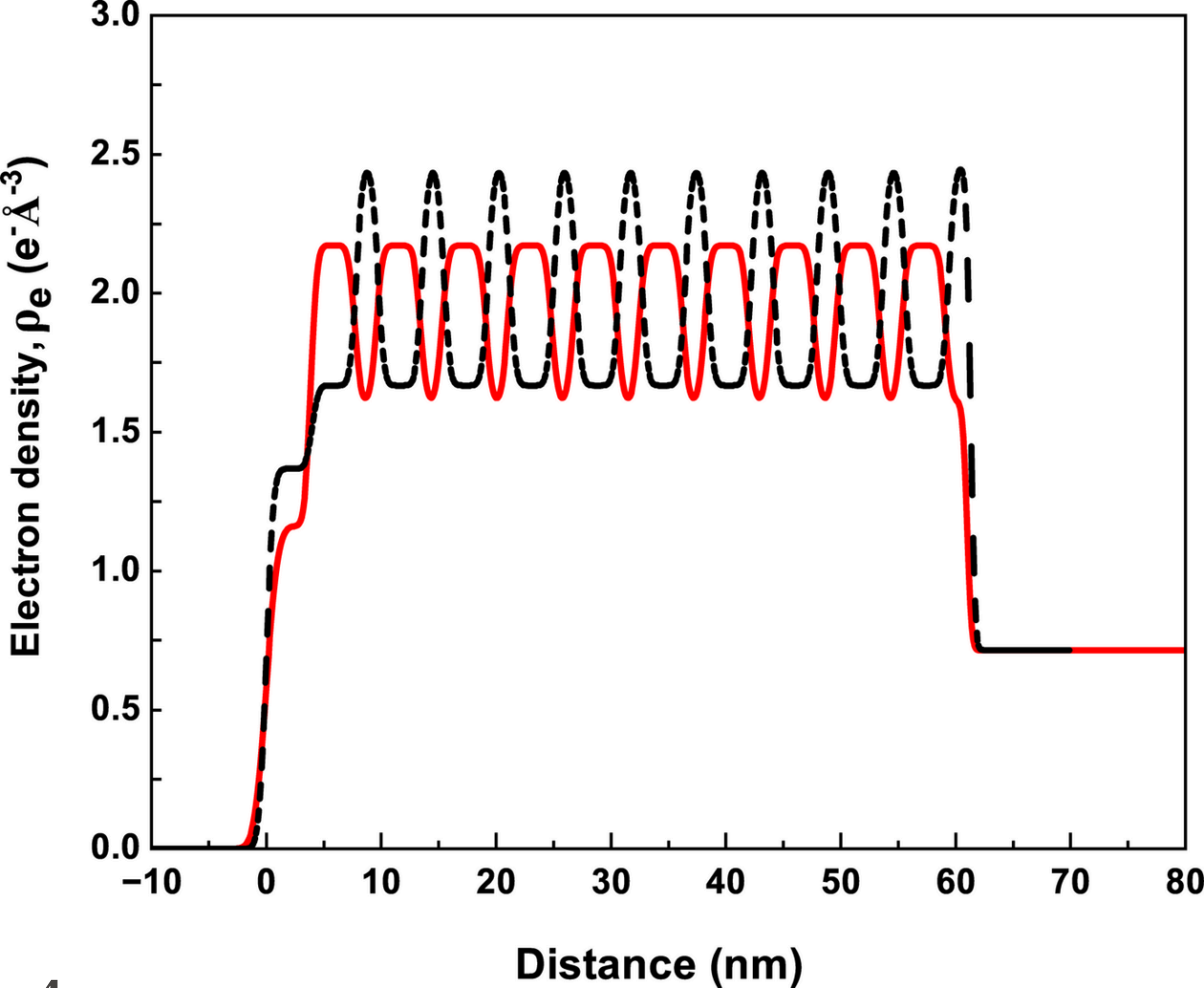
The electron density profile of Cu/Nb multilayers sample ML-2 obtained from the best of reflectivity curves near the Cu (continuous line) and Nb (dashed line) *K*-absorption edges.

The electron density profiles (ρ_e_) of the Cu/Nb multilayer sample ML-2 obtained from the best-fit models are shown in Fig. 4 ▸. The inclusion of a thin contamination layer and symmetric interfaces yields a satisfactory fit to the reflectivity data. As the incident photon energy approaches an absorption edge, the bound electrons begin to contribute significantly to the scattering process. This results in a pronounced change in the real dispersion term *f* ′(λ), which effectively reduces the free-electron contribution *f*^ *o*^(*q*_*z*_) of the resonant element. As a result, the effective electron density of the resonant atom decreases in the vicinity of its absorption edge. Near the Cu *K*-edge, Cu therefore appears to have a lower effective electron density than Nb, while near the Nb *K*-edge, Nb appears lower than Cu. This energy-dependent contrast variation can lead to an apparent inversion in the electron density profiles. Moreover, a single structural model provides satisfactory fits at both the Cu and Nb *K*-absorption edges, demonstrating the consistency of the fitting approach. The structural parameters obtained from the best-fit models shown in Fig. 3 ▸ are summarized in Table 2 ▸. The uncertainties in the fitted parameters were estimated by varying each parameter within 5% of χ^2^ from its minimum value. The layer thicknesses and interface roughness values obtained from the best-fit reflectivity curves at different energies agree within experimental uncertainty. The fitted electron densities are also consistent with literature values, except for the Cu layer in sample ML-1, where the deviation exceeds the fitting uncertainty near the Cu *K*-absorption edge.

Significant deviations of experimentally determined optical constants from the tabulated values reported by Henke *et al.* (1993 ▸) near absorption edges have been widely reported in the literature. Such deviations may arise from several factors, including (i) limited energy resolution in the vicinity of absorption edges, (ii) spectral contamination and (iii) restrictions in the measured energy range of the absorption coefficient, with the dispersion coefficient subsequently estimated using the Kramers–Kronig relation by extrapolation to infinite energy (Delmotte *et al.*, 2018 ▸; Rao *et al.*, 2010 ▸). In the present study, no significant deviations are observed in the estimated electron densities of Cu and Nb near the absorption edges. One factor contributing to the uncertainty in the estimated structural parameters is the uncertainty in the calibrated beamline energy, which is ultimately limited by the energy resolution of the beamline. Even a small error in the calibrated energy can lead to significant variations in the effective electron density of resonant elements close to their absorption edges.

**Figure 5:**
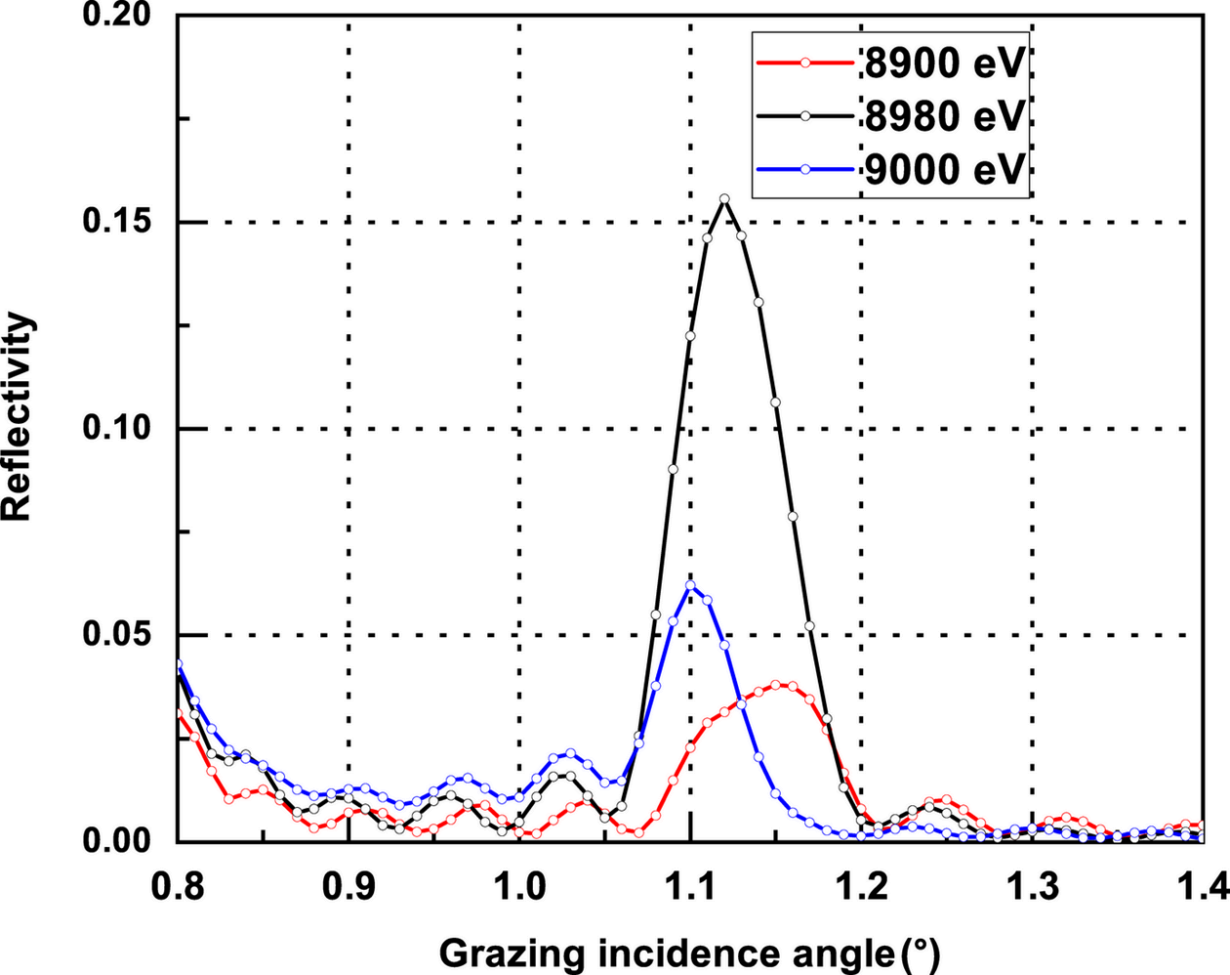
The measured first Bragg peak reflectance of Cu/Nb multilayer sample ML-1 in the vicinity of the Cu *K*-absorption edge (*E* = 8980 eV).

To illustrate this energy sensitivity, reflectivity measurements were carried out across the Cu *K*-absorption edge (*E* = 8980 eV). Fig. 5 ▸ shows the first-order Bragg peak reflectance of the Cu/Nb multilayer sample ML-1 near this edge. The reflectivity reaches a maximum at 8980 eV due to the highest electron density contrast and decreases as the energy deviates from this value. Specifically, the reflectivity decreases from ∼15% to 6% when the energy shifts from 8980 to 9000 eV, and from ∼15% to 4% when shifted from 8980 to 8900 eV, clearly demonstrating the strong influence of small energy variations on electron density contrast near absorption edges.

**Figure 6:**
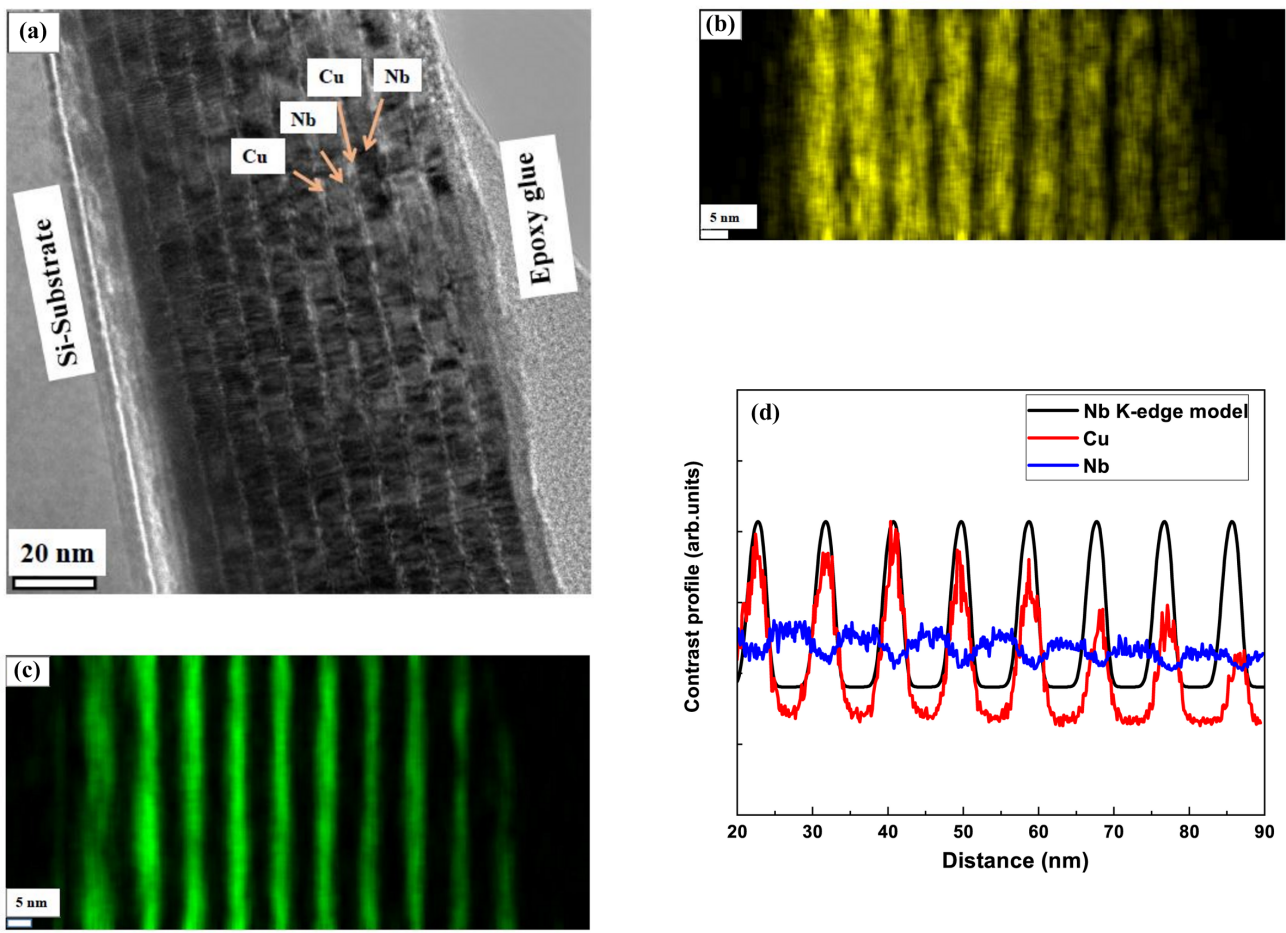
(*a*) CXTEM image, TEM–EDS elemental maps of (*b*) Nb and (*c*) Cu, and (*d*) reflectivity model curve near the Nb *K*-edge and EDS elemental profile of Cu/Nb multilayer sample ML-1.

The structural parameters of the Cu/Nb multilayers were independently evaluated using CXTEM. The CXTEM image of the Cu/Nb multilayer sample ML-1 is shown in Fig. 6 ▸(*a*). The image clearly reveals that the Cu and Nb layers are continuous and exhibit well defined sharp interfaces. The multilayer period and individual layer thicknesses were determined by extracting intensity profiles across the layers at different regions of the image. The average layer thicknesses estimated from the TEM analysis are 2.5 nm for Cu and 6.5 nm for Nb, resulting in a multilayer period of 9.0 nm. The multilayer period obtained from the TEM micrograph agrees well with the value derived from RXRR. However, slight variations are observed in the individual layer thicknesses, which can be attributed to the highly localized nature of TEM measurements and possible local thickness fluctuations.

Figs. 6 ▸(*b*) and 6 ▸(*c*) show the elemental distribution maps obtained from EDS corresponding to Fig. 6 ▸(*a*), further confirming the regular alternating arrangement of Cu and Nb layers. The EDS line profile, together with the electron density profile obtained from the best-fit reflectivity model near the Nb *K*-absorption edge, is presented in Fig. 6 ▸(*d*). The gradual compositional transitions observed in the EDS profile are attributed to interface roughness and are in good agreement with the reflectivity-based structural model. Overall, the TEM–EDS results corroborate the structural model assumed in the reflectivity analysis.

## 5. Conclusions

In summary, the conventional X-ray reflectivity technique using Cu *K*α radiation is insufficient for probing metallic multilayers with low electron density contrast. Tuning the incident energy to the absorption edge of constituent elements significantly enhances the contrast, enabling accurate determination of structural parameters. We demonstrated that, using resonant reflectivity, the layer thicknesses in Cu/Nb multilayers can be estimated non-destructively with sub-nanometre accuracy. This approach opens up new possibilities for tailoring the length-scale-dependent properties of low-contrast metallic multilayers.

## Figures and Tables

**Table 1:** The electron density contrast of selected metallic multilayers at Cu *K*α wavelength and *K*-absorption edges of respective elements

Multilayer combination (element-1 / element-2)	Δρ_e_/ρ_e_ (%)		
	At Cu *K*α	At *K*-absorption edge of element-1	At *K*-absorption edge of element-2
Co / Cr	11	36.6	102
Co / Cu	2.4	61.1	56.2
Co / Ni	1.9	62.8	48.1
Cu / Cr	13	32.0	106
Cu / Nb	0.9	48.8	52.7
Cu / Ni	0.4	57.6	45.7
Fe / Cr	6	52.1	78.9
Fe / Co	5.7	77.4	43.1
Fe / Cu	8	84.8	42.9
Nb / Ni	0.5	58.5	45.9

**Table 2:** Layer thickness (*d*), interface roughness (σ) and electron density (ρ_e_) values obtained from the best fit of RXRR curves shown in Fig. 3 ▸ The tabulated electron densities are shown in square brackets.

Sample name	Energy (eV)	*d*_Nb_ (nm)	*d*_Cu_ (nm)	ρ_Nb_ (e^−^ Å^−3^)	ρ_Cu_ (e^−^ Å^−3^)	σ_Cu-on-Nb_ (nm)	σ_Nb-on-Cu_ (nm)
ML-1	8980	6.28 ± 0.04	2.68 ± 0.04	2.30 ± 0.05 [2.26]	1.71 ± 0.05 [1.50]	0.70 ± 0.04	0.50 ± 0.07
	18900	6.33 ± 0.06	2.67 ± 0.07	1.93 ± 0.09 [1.87]	2.55 ± 0.08 [2.50]	0.63 ± 0.09	0.6 ± 0.1
ML-2	8980	3.80 ± 0.02	1.90 ± 0.02	2.15 ± 0.05 [2.26]	1.58 ± 0.05 [1.50]	0.43 ± 0.03	0.43 ± 0.02
	19000	3.85 ± 0.02	1.90 ± 0.02	1.66 ± 0.06 [1.67]	2.47 ± 0.05 [2.50]	0.44 ± 0.05	0.44 ± 0.05
